# Prognostic value of blood pressure and resting heart rate in patients with tricuspid regurgitation

**DOI:** 10.3389/fcvm.2022.937412

**Published:** 2022-08-03

**Authors:** Shuai Guo, Bin Zhang, Yunqing Ye, Zhe Li, Qingrong Liu, Zhenyan Zhao, Weiwei Wang, Zikai Yu, Haitong Zhang, Qinghao Zhao, Zhenya Duan, Junxing Lv, Erli Zhang, Bincheng Wang, Yanyan Zhao, Runlin Gao, Haiyan Xu, Yongjian Wu

**Affiliations:** ^1^Department of Cardiology, Fuwai Hospital, National Center for Cardiovascular Diseases, Chinese Academy of Medical Sciences and Peking Union Medical College, Beijing, China; ^2^Medical Research and Biometrics Center, Fuwai Hospital, National Center for Cardiovascular Diseases, Chinese Academy of Medical Sciences and Peking Union Medical College, Beijing, China

**Keywords:** tricuspid regurgitation, blood pressure, resting heart rate, prognosis, cohort study

## Abstract

**Background:**

The prognostic value of blood pressure (BP) and resting heart rate (RHR) in tricuspid regurgitation (TR) patients is unknown.

**Aims:**

This study aimed to investigate the associations of BP and RHR with all-cause mortality in patients with TR.

**Methods:**

A total of 2,013 patients with moderate or severe TR underwent echocardiography and BP measurement. The associations of routinely measured BP and RHR with 2-year all-cause mortality were analyzed.

**Results:**

The cohort had 45.9% male patients and a mean age of 62.5 ± 15.9 years. At the 2-year follow-up, 165 patient deaths had occurred. The risk of death decreased rapidly, negatively correlating with systolic blood pressure (SBP) up to 120 mmHg and diastolic blood pressure (DBP) up to 70 mmHg. For RHR, the risk increased in direct proportion, starting at 80 beats per min. After adjusting for age, sex, body mass index (BMI), diabetes, coronary heart disease, pulmonary hypertension, estimated glomerular filtration rate (eGFR), and NYHA class, SBP [hazard ratio (HR):0.89; 95% CI:0.823–0.957 per 10 mmHg increase; *P* =0.002], DBP (HR:0.8; 95% CI:0.714–0.908 per 10 mmHg increase; *P* < 0.001), and RHR (HR: 1.1; 95% CI: 1.022–1.175 per 10 beats per min increase; *P* = 0.011) were independently associated with all-cause mortality. These associations persisted after further adjustments for echocardiographic indices, medications, serological tests, and etiologies.

**Conclusion:**

In this cohort of patients with TR, routinely measured BP and RHR were associated with all-cause mortality independently. However, further large-scale, high-quality studies are required to validate our findings.

## Introduction

Tricuspid regurgitation (TR) is the most common tricuspid valve lesion (1). The prevalence of moderate to severe TR was estimated to be 2.6% in community populations over 65 years of age (2). Mild TR is generally considered benign, but moderate or severe TR can lead to right ventricular remodeling and dysfunction through increased right ventricular wall tension and adverse clinical outcomes (3, 4). Multiple studies have noted that the presence and severity of TR are associated with excess risk of mortality (5–8). A recent meta-analysis pointed out that the risk of all-cause mortality in patients with moderate or severe TR was twice as high as that in patients with no or mild TR, and this effect was independent of the pulmonary artery pressure and right ventricular function (9). Altogether, increasing evidence has indicated that TR is not only a lesion coexisting with other cardiac abnormalities but also a potential cause of adverse outcomes (10).

Compared with left-sided valvular heart disease, TR is often overlooked, and research focusing on the prognostic factors of tricuspid regurgitation is very limited. As symptoms and prognosis may improve with TR correction, it is vital to explore readily available prognostic factors to aid routine surveillance and clinical decisions for TR (11). The prognostic value of blood pressure (BP) and resting heart rate (RHR) has been confirmed in various diseases (12–14). However, to the best of our knowledge, no study has focused on the prognostic value of these parameters in patients with TR. Therefore, the study's main aim was to explore the prognostic importance of systolic blood pressure (SBP), diastolic blood pressure (DBP), and RHR in patients with TR.

## Methods

### Study population

The China valvular heart disease study (China-VHD study; NCT03484806) is a national, multicenter, prospective cohort study of patients with valvular heart disease. The study was conducted from April–June 2018 at 46 medical centers throughout mainland China. This cohort recruited 13,917 consecutive patients from inpatient wards and outpatient clinics, who had moderate or severe valvular heart disease as defined by echocardiography. In total, 2,147 patients with isolated moderate or severe native TR in the China-VHD cohort were included in this study. After excluding patients with peripheral arterial disease, infective endocarditis, and malignant tumor, and patients without baseline BP, RHR, or follow-up data, 2,013 patients remained (Supplementary materials). Ethical approval was obtained from the Ethics Committee of Fuwai Hospital, and the study protocol was approved by the institutional review board of the National Center for Cardiovascular Diseases of China. Written informed consent was obtained from all the participants present in this study.

### Clinical measurements

BP was measured by trained clinicians using a calibrated electronic sphygmomanometer with an appropriate cuff size, and this was done after the patient had settled for several minutes. RHR was taken in a sitting position along with BP.

### Echocardiography

A comprehensive 2-dimensional transthoracic echocardiography was performed on all patients using commercially available ultrasound systems. Echocardiograms were obtained following the standard protocol provided to the operators and reporters at the participating sites. Operators and reporters with more than five years of experience in cardiovascular ultrasound were selected. All operators and reporters received training before enrollment. The core laboratory at Fuwai Hospital verified the diagnostic accuracy and measurement consistency of the ultrasound images from each medical center. Operators were blinded to each other's results.

The dimensions of the left atrium and left ventricle were measured according to the recommendations of the American Society of Echocardiography and the European Association of Cardiovascular Imaging (15). Left ventricular systolic function was evaluated using the left ventricular ejection fraction measured with the biplane modified Simpson method. Significant TR was graded as moderate (central jet area 5-10 cm^2^, or vena contracta width <7 mm) and severe (central jet area >10 cm^2^, or flow reversal to the top of the right atrium or inferior vena cava, or vena contracta width ≥7 mm, or central jet TR-to-right atrium ratio >2/3, or regurgitant volume ≥45 mL/beat, or effective regurgitant orifice area ≥40 mm^2^).

Senior doctors determined the etiology of TR, combined with echocardiographic features, medical history, and surgical pathology reports (if provided). Primary or organic TR was caused by lesions of the leaflets or sub-valvular apparatus. Secondary or functional TR with intact leaflets and sub-valvular apparatus was caused by malcoaptation of the leaflets because of annular enlargement or right ventricular dilatation or dysfunction (16).

### Endpoints

The primary endpoint of this study was all-cause mortality. Since this study aimed to explore the risk of all-cause mortality in patients with TR under medical treatment, the follow-up started on the date of signing of the informed consent and was censored at the date of invasive tricuspid intervention, if performed during follow-up. Follow-up was conducted every 6 months for 2 years. Follow-up data were collected from outpatient clinics, medical records, or telephone interviews. The reported deaths and tricuspid interventions were reviewed and verified by investigators at each medical center. During enrollment, audits and quality controls were performed at randomly selected sites.

### Statistical analysis

Data are presented as mean ± *SD*, median [interquartile range (IQR)], or percentage. Differences between groups were evaluated using an unpaired student's *t*-test or *Wilcoxon rank-sum test* for continuous variables and chi-square test for categorical variables. Multiple imputations (random forest method) were used to impute the missing values.

Restricted cubic splines were used to evaluate the associations between BP and RHR and all-cause mortality. Subsequently, BP and RHR were analyzed as categorical and continuous variables. For categorical analysis, the patients were dichotomized according to the thresholds determined by the trend of restricted cubic splines and clinical significance. For continuous analysis, associations of SBP, DBP, and RHR with mortality were defined as a pressure increase of 10 mmHg or a heart rate increase of 10 beats per min (bpm). Survival curves were fitted using the Kaplan–Meier estimator. Hazard ratios (HRs) with 95% CIs were calculated using Cox proportional hazards analysis. The proportional hazard assumption was tested using Schoenfeld residual plots. Univariable Cox regression analysis was performed with relevant baseline characteristics to identify potential variables associated with the outcome. Variables with *P* < 0.05 in univariable Cox regression analysis were included in multivariable Cox regression analysis using different combinations of adjustments.

Multiple subgroup analyses were conducted to analyze the heterogeneity of the associations between hemodynamic parameters and the risk of death. Subgroup analyses were performed by including interactions between hemodynamic parameters and subgroup variables in the crude and adjusted Cox models. Since each etiology had a limited number of patients, in subgroup analyses, the etiology of TR was reclassified as organic (rheumatic, degenerative, and congenital), functional, and other.

Finally, C-statistics were calculated to evaluate the incremental prognostic value, the CIs for the changes in C-statistics were calculated with 1,000 bootstrap samples and *p*-values were calculated using the Z testing method. Furthermore, the net reclassification index (NRI) and integrated discrimination improvement (IDI) evaluated the incremental prognostic value of BP and RHR. Statistical analysis was performed with R version 4.0.5 (R Foundation for Statistical Computing, Vienna, Austria) using the “compareGroups,” “survival,” “rms,” “mice,” “survIDINRI,” and “CsChange” packages.

## Results

### Baseline characteristics

In our study, 1,774 (88.13%) patients from inpatient wards and 239 (11.87%) from outpatient clinics were enrolled. The baseline characteristics of 2,013 patients are summarized in Table 1. The mean age was 62.5 ± 15.9 years, and 924 (45.9%) were males. Average SBP, DBP, and RHR were 125 ± 21.1 mmHg, 75.2 ± 12.9 mmHg, and 78.7 ± 19.2 bpm, respectively. Most patients (83.0%) were symptomatic, and 32.7% were NYHA class III/IV. Among these patients, 645 (32.0%) had severe TR. Among the 2,013 patients, the most common etiology was functional TR (55.7%), followed by degenerative TR (17.4%) and congenital TR (12.0%). During the follow-up period, 192 (9.54%) patients underwent tricuspid valve intervention.

**Table 1:** Baseline characteristics of the study population.

	**Total**	**SBP**		***p*-Value**	**DBP**		***p*-Value**	**RHR**		***p*-Value**
	**(*N =* 2,013)**	**≥120 mmHg**	** <120 mmHg**		**≥70 mmHg**	** <70 mmHg**		**≥80 bpm**	** <80 bpm**	
		**(*N =* 1,218)**	**(*N =* 795)**		**(*N =* 1,392)**	**(*N =* 621)**		**(*N =* 885)**	**(*N =* 1,128)**	
Age, yrs	62.5 ± 15.9	66.1 ± 14.0	57.1 ± 17.1	<0.001	63.0 ± 15.4	61.3 ± 17.0	0.032	60.7 ± 16.5	64.0 ± 15.2	<0.001
Male	924 (45.9%)	586 (48.1%)	338 (42.5%)	0.016	669 (48.1%)	255 (41.1%)	0.004	422 (47.7%)	502 (44.5%)	0.169
BMI, kg/m^2^	23.2 ± 3.91	23.7 ± 3.70	22.6 ± 4.11	<0.001	23.5 ± 3.82	22.6 ± 4.02	<0.001	23.4 ± 4.36	23.1 ± 3.50	0.120
Current smoker	240 (11.9%)	138 (11.3%)	102 (12.8%)	0.345	170 (12.2%)	70 (11.3%)	0.598	116 (13.1%)	124 (11.0%)	0.166
SBP, mmHg	125 ± 21.1	138 ± 16.3	106 ± 9.46	0.000	130 ± 19.8	114 ± 19.3	<0.001	124 ± 21.3	126 ± 21.0	0.203
DBP, mmHg	75.2 ± 12.9	79.8 ± 12.8	68.2 ± 9.38	<0.001	81.3 ± 10.2	61.5 ± 5.94	<0.001	78.2 ± 13.4	72.9 ± 12.0	<0.001
Hypertension	837 (41.6%)	654 (53.7%)	183 (23.0%)	<0.001	644 (46.3%)	193 (31.1%)	<0.001	346 (39.1%)	491 (43.5%)	0.050
Resting heart rate, bpm	78.7 ± 19.2	78.1 ± 19.2	79.6 ± 19.0	0.091	80.8 ± 19.5	73.8 ± 17.3	<0.001	94.5 ± 16.7	66.3 ± 9.29	<0.001
Diabetes	285 (14.2%)	211 (17.3%)	74 (9.31%)	<0.001	204 (14.7%)	81 (13.0%)	0.374	123 (13.9%)	162 (14.4%)	0.817
Coronary artery disease	517 (25.7%)	372 (30.5%)	145 (18.2%)	<0.001	371 (26.7%)	146 (23.5%)	0.151	199 (22.5%)	318 (28.2%)	0.004
Cardiomyopathy	112 (5.56%)	59 (4.84%)	53 (6.67%)	0.100	77 (5.53%)	35 (5.64%)	1.000	55 (6.21%)	57 (5.05%)	0.303
Atrial fibrillation	755 (37.5%)	487 (40.0%)	268 (33.7%)	0.005	560 (40.2%)	195 (31.4%)	<0.001	359 (40.6%)	396 (35.1%)	0.014
Pulmonary hypertension	1,186 (58.9%)	701 (57.6%)	485 (61.0%)	0.135	805 (57.8%)	381 (61.4%)	0.151	535 (60.5%)	651 (57.7%)	0.232
Previous PCI	168 (8.35%)	109 (8.95%)	59 (7.42%)	0.259	108 (7.76%)	60 (9.66%)	0.181	59 (6.67%)	109 (9.66%)	0.020
Previous CABG	49 (2.43%)	39 (3.20%)	10 (1.26%)	0.009	37 (2.66%)	12 (1.93%)	0.413	6 (0.68%)	43 (3.81%)	<0.001
Previous valvular intervention	147 (7.30%)	80 (6.57%)	67 (8.43%)	0.139	96 (6.90%)	51 (8.21%)	0.339	73 (8.25%)	74 (6.56%)	0.174
Pacemaker/ICD implantation	164 (8.15%)	111 (9.11%)	53 (6.67%)	0.060	107 (7.69%)	57 (9.18%)	0.297	37 (4.18%)	127 (11.3%)	<0.001
NYHA class ≥III	659 (32.7%)	362 (29.7%)	297 (37.4%)	<0.001	452 (32.5%)	207 (33.3%)	0.742	362 (40.9%)	297 (26.3%)	<0.001
Severe TR	645 (32.0%)	342 (28.1%)	303 (38.1%)	<0.001	398 (28.6%)	247 (39.8%)	<0.001	282 (31.9%)	363 (32.2%)	0.918
Symptoms	1,670 (83.0%)	1,010 (82.9%)	660 (83.0%)	1.000	1,155 (83.0%)	515 (82.9%)	1.000	746 (84.3%)	924 (81.9%)	0.177
eGFR, ml/min/1.73 m^2^	78.3 ± 23.9	75.9 ± 23.0	81.9 ± 25.0	<0.001	78.6 ± 22.9	77.6 ± 26.1	0.418	78.9 ± 24.4	77.8 ± 23.6	0.316
Hemoglobin, g/L	132 ± 24.2	131 ± 23.5	133 ± 25.4	0.202	134 ± 23.0	127 ± 26.0	<0.001	134 ± 25.3	130 ± 23.2	0.001
Albumin, g/L	39.1 ± 5.44	39.2 ± 5.32	38.8 ± 5.63	0.099	39.2 ± 5.27	38.7 ± 5.80	0.045	38.7 ± 5.66	39.4 ± 5.25	0.009
ALT, U/L	18.1 [13.0;28.2]	18.6 [13.0;28.1]	18.0 [13.0;28.2]	0.382	19.0 [13.0;28.6]	18.0 [12.5;28.0]	0.640	19.4 [13.9;31.0]	17.4 [12.0;26.0]	<0.001
Total bilirubin, μmol/L	15.4 [11.1;22.5]	14.8 [10.5;20.6]	17.4 [12.0;25.5]	<0.001	15.3 [11.0;22.3]	15.8 [11.2;23.2]	0.325	16.4 [11.7;24.7]	14.7 [10.7;20.7]	<0.001
LAi, mm/m^2^	25.4 ± 5.77	25.5 ± 5.59	25.1 ± 6.02	0.118	25.2 ± 5.66	25.6 ± 6.00	0.167	25.4 ± 6.01	25.4 ± 5.57	0.906
LVEDDi, mm/m^2^	28.1 ± 5.16	28.4 ± 4.73	27.7 ± 5.74	0.009	28.0 ± 4.86	28.3 ± 5.78	0.346	27.8 ± 5.63	28.3 ± 4.75	0.022
LVEF, %	56.9 ± 11.5	57.0 ± 11.0	56.8 ± 12.2	0.698	56.7 ± 11.5	57.6 ± 11.3	0.104	55.6 ± 12.2	58.0 ± 10.7	<0.001
AAO, mm	31.5 ± 5.20	31.9 ± 4.90	30.8 ± 5.58	<0.001	31.7 ± 4.96	31.1 ± 5.71	0.037	31.4 ± 5.14	31.5 ± 5.26	0.604
Etiology				<0.001			0.017			<0.001
Rheumatic	145 (7.88%)	79 (7.14%)	66 (8.99%)		95 (7.47%)	50 (8.80%)		86 (10.4%)	59 (5.80%)	
Degenerative	320 (17.4%)	218 (19.7%)	102 (13.9%)		215 (16.9%)	105 (18.5%)		122 (14.8%)	198 (19.5%)	
Congenital	221 (12.0%)	102 (9.22%)	119 (16.2%)		135 (10.6%)	86 (15.1%)		105 (12.8%)	116 (11.4%)	
Functional	1,025 (55.7%)	628 (56.8%)	397 (54.1%)		738 (58.0%)	287 (50.5%)		451 (54.8%)	574 (56.4%)	
Other	129 (7.01%)	79 (7.14%)	50 (6.81%)		89 (7.00%)	40 (7.04%)		59 (7.17%)	70 (6.88%)	
ACEI/ARB	740 (36.8%)	533 (43.8%)	207 (26.0%)	<0.001	546 (39.2%)	194 (31.2%)	0.001	307 (34.7%)	433 (38.4%)	0.097
Beta blocker	967 (48.0%)	638 (52.4%)	329 (41.4%)	<0.001	676 (48.6%)	291 (46.9%)	0.510	451 (51.0%)	516 (45.7%)	0.023
Diuretic	1,191 (59.2%)	667 (54.8%)	524 (65.9%)	<0.001	794 (57.0%)	397 (63.9%)	0.004	600 (67.8%)	591 (52.4%)	<0.001
Tricuspid intervention	192 (9.54%)	103 (8.46%)	89 (11.2%)	0.049	120 (8.62%)	72 (11.6%)	0.044	85 (9.60%)	107 (9.49%)	0.989

Values are mean ± SD, n (%) or median [interquartile range]. AAO, ascending aorta; ACEI, angiotensin converting enzyme inhibitors; ALT, alanine aminotransferase; ARB, angiotensin receptor blocker; BMI, body mass index; CABG, coronary artery bypass grafting; DBP, diastolic blood pressure; eGFR, estimated glomerular filtration rate; ICD, implantable cardiac defibrillator; LAi, indexed left atrial end-diastolic dimension; LVEF, left ventricular ejection fraction; LVEDDi, indexed left ventricular end-diastolic dimension; PCI, percutaneous coronary intervention; RHR, resting heart rate; SBP, systolic blood pressure; TR, tricuspid regurgitation.

### BP/RHR cutoffs for predicting all-cause mortality

**Figure 1:**
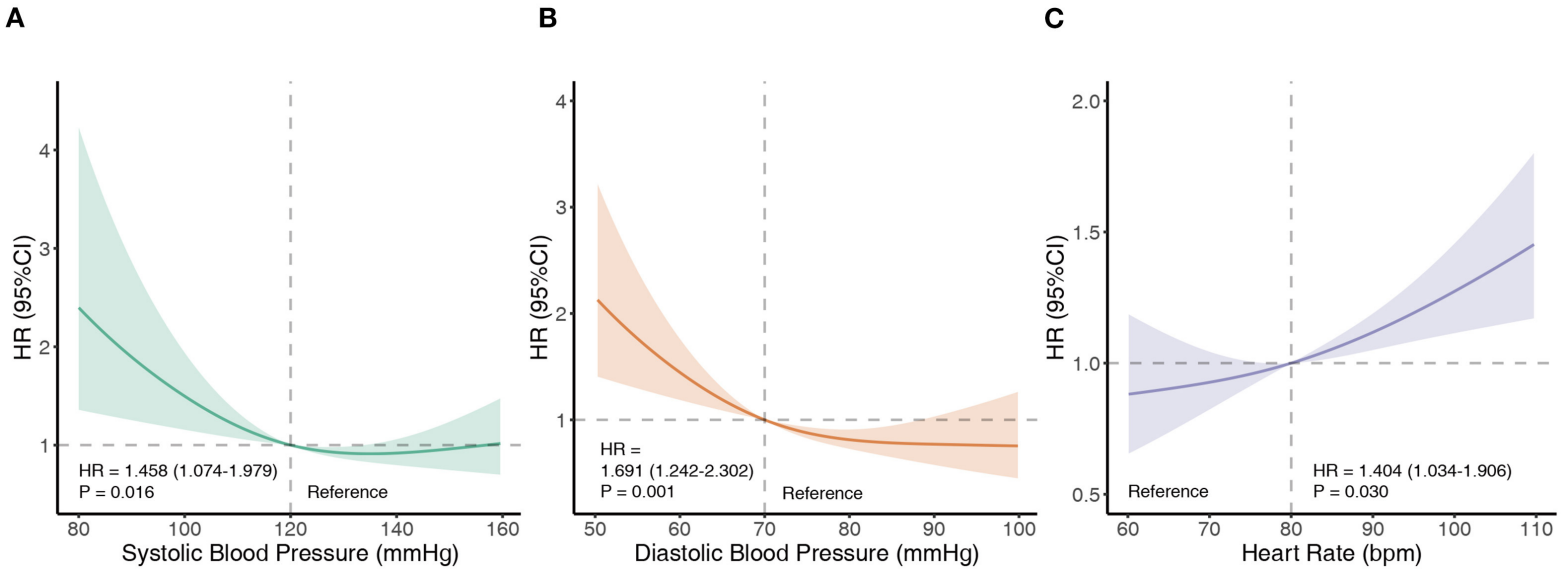
Associations of SBP, DBP, and RHR with all-cause mortality. Restricted cubic splines demonstrate associations of SBP **(A)**, DBP **(B)**, and RHR **(C)** with all-cause mortality. The splines are presented as hazard ratios (HRs) with 95% confidence intervals. Bpm, beats per minute; DBP, diastolic blood pressure; HR, hazard ratio; RHR, resting heart rate; SBP, systolic blood pressure.

At the 2-year follow-up, 165 patient deaths had occurred. The overall survival at 1 year and 2 years was 93.18 ± 0.68% and 89.29 ± 0.89%, respectively. Restricted cubic spline analyses were performed to investigate the trend in associations of SBP, DBP, and RHR with all-cause mortality (Figure 1). The *p-*values for the nonlinearity of each spline were 0.0237, 0.0677, and 0.4804, respectively. For SBP and DBP, the splines of the HR initialized with a plateau and then demonstrated a significant increase with a decrease in BP. Moreover, a significant increase in HR with an increase in RHR was observed. Based on the restricted cubic splines, thresholds of 120 mmHg for SBP, 70 mmHg for DBP, and 80 bpm for RHR were used to dichotomize the TR populations.

**Figure 2:**
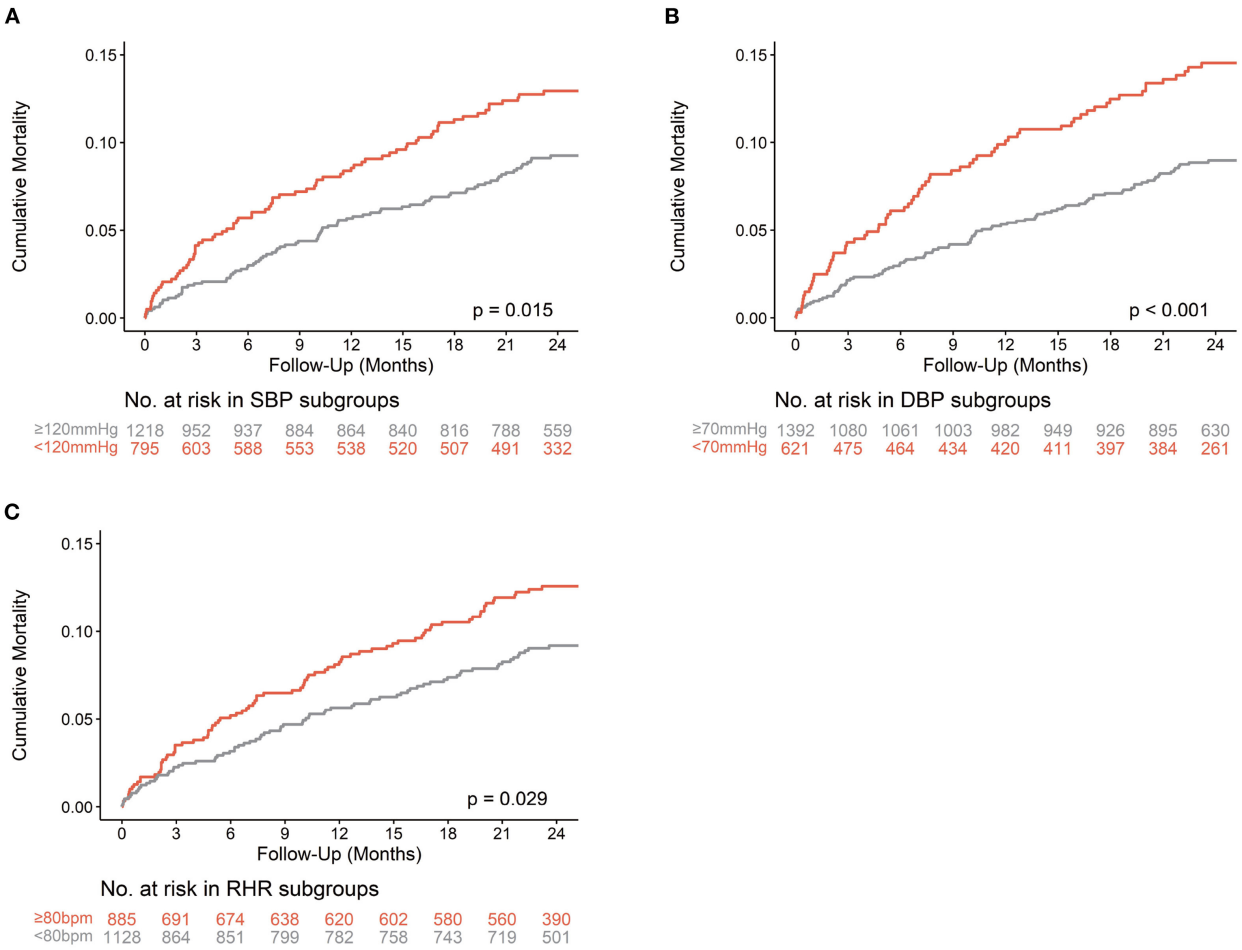
Kaplan-Meier curves of all-cause Mortality. The Kaplan**-**Meier curves of all-cause mortality are shown according to thresholds of SBP **(A)**, DBP **(B)**, and RHR **(C)** in patients with TR. Bpm, beats per minute; DBP, diastolic blood pressure; RHR, resting heart rate; SBP, systolic blood pressure.

Kaplan–Meier curves and log-rank tests indicated that low SBP (Kaplan–Meier log-rank *p* = 0.015), low DBP (Kaplan–Meier log-rank *P* < 0.001), and high RHR (Kaplan–Meier log-rank *p* = 0.029) in patients with TR were significantly associated with worse survival (Figure 2).

Remarkably, compared with patients with SBP ≥120 mmHg and DBP ≥70 mmHg, those with lower SBP (<120 mmHg) and DBP (<70 mmHg) were younger, more likely to be female, had a lower body mass index (BMI), less atrial fibrillation, more severe TR, more frequent TR intervention, and increased use of diuretics. From the perspective of RHR, compared to patients with RHR <80 bpm, patients with higher RHR (≥80 bpm) were younger, had less coronary heart disease, more AF, less previous percutaneous coronary intervention (PCI), and coronary artery bypass grafting (CABG), worse NYHA class, higher hemoglobin, and total bilirubin, lower albumin, lower indexed left ventricular end-diastolic dimension (LVEDDi) and left ventricular ejection fraction (LVEF), and increased use of beta-blockers and diuretics.

### Prognostic value

Univariable Cox regression analysis revealed that age, BMI, SBP, DBP, RHR, diabetes, coronary heart disease, pulmonary hypertension, estimated glomerular filtration rate (eGFR), NYHA class, severe TR, symptoms, hemoglobin, albumin, total bilirubin, LVEDDi, LVEF, etiology, and use of beta-blockers and diuretics were potential predictors of all-cause mortality (Supplementary materials). After adjusting for age, sex, BMI, diabetes, coronary heart disease, pulmonary hypertension, eGFR, and NYHA class, SBP (HR per 10 mmHg increase, 0.89; 95% CI:0.823–0.957; *p* = 0.002), DBP (HR per 10 mmHg increase, 0.80; 95% CI:0.714–0.908; *P* < 0.001), and RHR (HR per 10 bpm increase, 1.10; 95% CI: 1.022–1.175; *p* = 0.011) were independently associated with all-cause mortality. The associations remained consistent after further separate adjustments for echocardiographic indices, medications, serological findings, and TR etiologies (Table 2). After the same adjustments in the combined models of BP and RHR, the statistical significance remained (Table 3).

**Table 2:** Associations of SBP, DBP and RHR With All-Cause Mortality.

	**Separate models for all-cause mortality; *N =* 2,013, 165 deaths**					
	**Baseline SBP (per 10 mmHg increase)**		**Baseline DBP (per 10 mmHg increase)**		**Baseline RHR (per 10 bpm increase)**	
	**HR (95% CI)**	***p*-Value**	**HR (95% CI)**	***p*-Value**	**HR (95% CI)**	***p*-Value**
Univariate Cox analysis	0.93 (0.860–0.998)	0.044	0.80 (0.709–0.907)	<0.001	1.12 (1.047–1.193)	0.001
Model 1. + Age, Sex, BMI, Diabetes, CAD, PH, NYHAc, eGFR	0.89 (0.823–0.957)	0.002	0.80 (0.714–0.908)	<0.001	1.10 (1.022–1.175)*	0.011
Model 2. Model 1+ Severe TR, LVEF, LVEDDi	0.90 (0.833–0.971)	0.007	0.80 (0.709–0.903)	<0.001	1.09 (1.013–1.169)	0.021
Model 3. Model 1+ β blocker, Diuretic	0.89 (0.823–0.958)	0.002	0.80 (0.713–0.905)	<0.001	1.08 (1.009–1.163)	0.027
Model 4. Model 1+ Hb, Alb, Tbil	0.91 (0.839–0.976)	0.010	0.84 (0.743–0.955)	0.007	1.10 (1.026–1.184)	0.008
Model 5. Model 1+ Etiology	0.86 (0.790–0.936)	<0.001	0.78 (0.680–0.888)	<0.001	1.12 (1.035–1.203)	0.004

AF, atrial fibrillation; Alb, albumin; BMI, body mass index; CAD, coronary heart disease; DBP, diastolic blood pressure; eGFR, estimated glomerular filtration rate; Hb, hemoglobin; LVEDDi, indexed left ventricular end-diastolic dimension; LVEF, left ventricular ejection fraction; PH, pulmonary hypertension; RHR, resting heart rate; SBP, systolic blood pressure; Tbil, total bilirubin; TR, tricuspid regurgitation. *For RHR, model 1 further adjusted for atrial fibrillation.

**Table 3:** Combined models of BPs and RHR for all-cause mortality.

**Combined models of SBP and RHR for all-cause mortality; *N =* 2013, 165 deaths**				
	**Baseline SBP (per 10 mmHg increase)**		**Baseline RHR (per 10 bpm increase)**	
	**HR (95% CI)**	***p*** **-Value**	**HR (95% CI)**	***p*** **-Value**
Model 1. + Age, Sex, BMI, Diabetes, CAD, PH, AF, NYHAc, eGFR	0.89 (0.826–0.958)	0.002	1.09 (1.018–1.171)	0.014
Model 2. Model 1 + Severe TR, LVEF, LVEDDi	0.90 (0.833–0.969)	0.005	1.09 (1.013–1.170)	0.020
Model 3. Model 1 + β blocker, Diuretic	0.89 (0.822–0.955)	0.002	1.08 (1.007–1.160)	0.031
Model 4. Model 1 + Hb, Alb, Tbil	0.91 (0.841–0.975)	0.009	1.10 (1.023–1.180)	0.010
Model 5. Model 1 + Etiology	0.86 (0.792–0.934)	<0.001	1.11 (1.033–1.202)	0.005
**Combined models of DBP and RHR for all-cause mortality;** ***N** =* **2,013, 165 deaths**				
	**Baseline DBP (per 10 mmHg increase)**		**Baseline RHR (per 10 bpm increase)**	
	**HR (95% CI)**	***p*** **-Value**	**HR (95% CI)**	***p*** **-Value**
Model 1. + Age, Sex, BMI, Diabetes, CAD, PH, AF, NYHAc, eGFR	0.76 (0.671–0.861)	<0.001	1.15 (1.070–1.232)	<0.001
Model 2. Model 1 + Severe TR, LVEF, LVEDDi	0.76 (0.667–0.859)	<0.001	1.14 (1.064–1.230)	<0.001
Model 3. Model 1 + β blocker, Diuretic	0.76 (0.672–0.860)	<0.001	1.14 (1.060–1.222)	<0.001
Model 4. Model 1 + Hb, Alb, Tbil	0.80 (0.705–0.912)	0.001	1.14 (1.062–1.227)	<0.001
Model 5. Model 1 + Etiology	0.72 (0.627–0.826)	<0.001	1.18 (1.096–1.273)	<0.001

AF, atrial fibrillation; Alb, albumin; BMI, body mass index; CAD, coronary heart disease; DBP, diastolic blood pressure; eGFR, estimated glomerular filtration rate; Hb, hemoglobin; LVEDDi, indexed left ventricular end-diastolic dimension; LVEF, left ventricular ejection fraction; PH, pulmonary hypertension; RHR, resting heart rate; SBP, systolic blood pressure; Tbil, total bilirubin; TR, tricuspid regurgitation.

### Subgroup analyses

For SBP and DBP, the risks of mortality were similar across several relevant subgroups to the main findings (all HRs <1), and there were no significant interactions between BP and any of the subgroups. However, for RHR, statistically significant interactions were identified across the etiology and beta-blocker subgroups. A higher risk of mortality with increasing RHR was found in organic (HR per 10 bpm increase, 1.18; 95% CI: 1.03–1.35; *p* =0.017) and other etiologies (HR per 10 bpm increase, 1.56; 95% CI: 1.24–1.95; *P* < 0.001) of TR. Furthermore, for the subgroup not taking beta-blockers, the prognostic value of RHR for all-cause mortality remained significant (HR per 10 bpm increase, 1.23; 95% CI: 1.09–1.39; *P* < 0.001) (Tables 4–6).

**Table 4:** Subgroup analyses of SBP in clinically meaningful subsets.

**Groups**	**Number of patients**	**Events**	**Crude**		**p for interaction**	**Adjusted** ^*^		**p for interaction**
			**HR (95%CI)**	***p*-value**		**HR (95%CI)**	***p*-value**	
Etiology					0.442			0.560
Organic TR	686	51	0.92 (0.80–1.05)	0.229		0.84 (0.72–0.98)	0.031	
Functional TR	1,025	78	0.91 (0.82–1.01)	0.089		0.91 (0.82–1.01)	0.064	
Other	129	13	0.73 (0.51–1.04)	0.078		0.78 (0.56–1.09)	0.141	
NYHAc					0.490			0.619
NYHA < III	1,300	54	0.99 (0.87–1.12)	0.846		0.90 (0.78–1.04)	0.168	
NYHA≥III	548	111	0.93 (0.86–1.02)	0.126		0.87 (0.78–0.96)	0.007	
LVEF					0.708			0.732
LVEF <50	289	55	0.93 (0.84–1.01)	0.100		0.90 (0.78–1.03)	0.111	
LVEF≥50	1,559	110	0.95 (0.84–1.08)	0.445		0.87 (0.78–0.97)	0.011	
ACEI/ARB					0.130			0.428
Yes	740	61	0.99 (0.88–1.10)	0.807		0.92 (0.81–1.06)	0.249	
No	1,273	104	0.88 (0.79–0.97)	0.013		0.86 (0.77–0.97)	0.011	
Diuretic					0.797			0.490
Yes	1,191	135	0.95 (0.87–1.03)	0.186		0.87 (0.79–0.95)	0.003	
No	822	30	0.97 (0.82–1.16)	0.751		0.93 (0.77–1.13)	0.488	

* Adjusted for age, sex, BMI, diabetes, CAD, PH, eGFR, severe TR, LVEF, LVEDDi, NYHAc, and etiology when they were not the strata variables. BMI, body mass index; CAD, coronary heart disease; eGFR, estimated glomerular filtration rate; LVEDDi, indexed left ventricular end-diastolic dimension; LVEF, left ventricular ejection fraction; PH, pulmonary hypertension; SBP, systolic blood pressure; TR, tricuspid regurgitation.

**Table 5:** Subgroup analyses of DBP in Clinically Meaningful Subsets.

**Groups**	**Number of Patients**	**Events**	**Crude**		**p for interaction**	**Adjusted** ^*^		**p for interaction**
			**HR (95%CI)**	***p*-value**		**HR (95%CI)**	***p*-value**	
Etiology					0.812			0.709
Organic TR	686	51	0.81 (0.64–1.03)	0.091		0.78 (0.61–0.99)	0.045	
Functional TR	1,025	78	0.76 (0.63–0.90)	0.002		0.78 (0.66–0.92)	0.003	
Other	129	13	0.86 (0.55–1.35)	0.512		0.95 (0.61–1.46)	0.808	
NYHAc					0.276			0.074
NYHA < III	1,300	54	0.90 (0.72–1.12)	0.341		0.95 (0.75–1.20)	0.658	
NYHA≥III	548	111	0.78 (0.68–0.89)	<0.001		0.73 (0.62–0.86)	<0.001	
LVEF					0.521			0.241
LVEF <50	289	55	0.82 (0.68–0.99)	0.036		0.87 (0.71–1.07)	0.196	
LVEF≥50	1,559	110	0.76 (0.65–0.89)	<0.001		0.74 (0.63–0.88)	<0.001	
ACEI/ARB					0.434			0.667
Yes	740	61	0.85 (0.70–1.02)	0.077		0.77 (0.61–0.97)	0.024	
No	1,273	104	0.76 (0.65–0.90)	0.002		0.82 (0.69–0.97)	0.018	
Diuretic					0.198			0.805
Yes	1,191	135	0.85 (0.75–0.96)	0.012		0.80 (0.69–0.92)	0.002	
No	822	30	0.68 (0.49–0.93)	0.017		0.76 (0.53–1.09)	0.131	

* Adjusted for age, sex, BMI, diabetes, CAD, PH, eGFR, severe TR, LVEF, LVEDDi, NYHAc, and etiology when they were not the strata variables. BMI, body mass index; CAD, coronary heart disease; DBP, diastolic blood pressure; eGFR, estimated glomerular filtration rate; LVEDDi, indexed left ventricular end-diastolic dimension; LVEF, left ventricular ejection fraction; PH, pulmonary hypertension; TR, tricuspid regurgitation.

**Table 6:** Subgroup analyses of RHR in Clinically Meaningful Subsets.

**Groups**	**Number of patients**	**Events**	**Crude**		**p for interaction**	**Adjusted** ^*^		**p for interaction**
			**HR (95%CI)**	***p*-value**		**HR (95%CI)**	***p*-value**	
Etiology					0.034			0.005
Organic TR	686	51	1.18 (1.04–1.32)	0.007		1.18 (1.03–1.35)	0.017	
Functional TR	1,025	78	1.08 (0.98–1.19)	0.129		1.03 (0.93–1.14)	0.574	
Other	129	13	1.51 (1.20–1.89)	<0.001		1.56 (1.24–1.95)	<0.001	
NYHAc					0.068			0.107
NYHA < III	1,300	54	1.15 (1.03–1.28)	0.011		1.21 (1.08–1.37)	0.001	
NYHA≥III	548	111	1.01 (0.92–1.10)	0.908		1.07 (0.98–1.17)	0.153	
LVEF					0.437			0.680
LVEF <50	289	55	1.06 (0.94–1.18)	0.335		1.09 (0.95–1.24)	0.212	
LVEF≥50	1,559	110	1.12 (1.03–1.21)	0.009		1.13 (1.03–1.23)	0.011	
β-blocker					0.018			0.040
Yes	967	96	1.05 (0.96;1.14)	0.267		1.05 (0.95–1.16)	0.379	
No	1,046	69	1.26 (1.11;1.41)	<0.001		1.23 (1.09–1.39)	<0.001	

* Adjusted for age, atrial fibrillation, sex, BMI, diabetes, CAD, PH, eGFR, severe TR, LVEF, LVEDDi, NYHAc, and etiology when they were not the strata variables. BMI, body mass index; CAD, coronary heart disease; eGFR, estimated glomerular filtration rate; LVEDDi, indexed left ventricular end-diastolic dimension; LVEF, left ventricular ejection fraction; PH, pulmonary hypertension; RHR, resting heart rate; TR, tricuspid regurgitation.

### Incremental prognostic value

A base model including age, sex, BMI, diabetes, coronary heart disease, pulmonary hypertension, eGFR, symptoms, severe TR, LVEF, and LVEDDi was constructed to evaluate the incremental value of BP and RHR. The addition of SBP, DBP, and RHR significantly improved the predictive capacity of the model (C-statistic difference = 0.027, *p* = 0.02; IDI = 0.021, *p* = 0.004; NRI = 0.179, *p* = 0.006) (Supplementary materials).

## Discussion

This national, multicenter, prospective cohort study revealed the prognostic value of SBP, DBP, and RHR in patients with TR. The primary findings of this study were as follows: (1) routinely measured SBP, DBP, and RHR were significant predictors of all-cause mortality in patients with TR, independent of echocardiographic indices, medications, serological findings, and etiologies. (2) Reverse relationships were observed among SBP, DBP, and all-cause mortality. For SBP, there was a non-linear relationship and the risk of all-cause mortality increased when SBP was <120 mmHg. As for DBP, a linear relationship was observed, and the risk of mortality began to increase when DBP was <70 mmHg. (3) RHR was positively associated with all-cause mortality, with excessive risk increasing above 80 bpm.

### Prognosis and BP in TR

Current clinical guidelines for the management of TR focus on the etiology, severity, symptoms, right ventricular or tricuspid annular size, pulmonary artery pressure, and ventricular function (17, 18). Baseline BP and RHR have not received adequate attention in the management of TR. However, in this study, we found that in patients with TR, the risk of all-cause mortality did not significantly change when SBP was ≥ 120 mmHg (120–160 mmHg). When the SBP was <120 mmHg, the risk of all-cause mortality significantly increased. A similar trend was also observed in DBP; there was no significant change in the risk of all-cause mortality when the DBP was between 70 and 100 mmHg, but it increased rapidly when the DBP was <70 mmHg. Therefore, hypotension in patients with TR should not be regarded as a clinically irrelevant sign but should be regarded as a significant prognostic marker and considered in clinical decision-making. Current hypertension guidelines and randomized controlled trials recommend intensive BP-lowering therapy; however, our study suggests that very low SBP or DBP should be avoided in the management of TR (19, 20).

Although patients with SBP <120 mmHg were relatively young and had low proportions of diabetes, coronary heart disease, and atrial fibrillation, these patients had severe TR, high proportions of NYHA class III-IV, and high total bilirubin. In addition, the use of diuretics was more common in these patients, as was the rate of invasive tricuspid valve intervention during follow-up. Similarly, patients with DBP <70 mmHg were relatively young, and there were more patients with severe TR, high rates of diuretic use, and invasive tricuspid intervention during follow-up.

When deciphering the hemodynamic characteristics of TR in the context of existing studies, potential explanations may address the associations of low SBP and low DBP with adverse outcomes in these patients. In our study, patients with low SBP and low DBP were characterized by high serum total bilirubin levels, which is a marker for congestive hepatopathy caused by TR-related backward failure (21, 22). This cardio-hepatic interaction may lead to disequilibrium of vasoactive substances and subsequently lead to a decrease in systemic vascular resistance (23). Ultimately, a decrease in BP was observed, especially in DBP. Coronary perfusion occurs during diastole, and a decrease in DBP may cause coronary hypoperfusion and myocardial ischemia, leading to adverse outcomes (24).

### Prognosis and RHR in TR

Consistent with previous studies, this study noted that a higher RHR was also associated with adverse outcomes in patients with TR (12, 25). In our study cohort, patients with an RHR ≥80 bpm had worse NYHA class, lower LVEF, and more use of beta-blockers and diuretics than those with RHR <80 bpm. Mechanistically, the cardio-hepatic interaction may lead to splanchnic vasodilatation, resulting in relative hypovolemia and glomerular hypoperfusion (26). Subsequent compensatory activation of the renin-angiotensin-aldosterone system results in water and sodium retention, further deteriorating the volume overload in the splanchnic vascular bed (27). Afterwards, the blood reserve in the splanchnic vascular bed is diverted to the central circulation due to increased sympathetic tone (28–30). Ultimately, the diversion of splanchnic volume overload and decrease in systemic vascular resistance leads to an increase in preload and a decrease in afterload. Combining these factors increases the RHR, myocardial oxygen consumption, and ventricular burden, leading to adverse outcomes.

### Subgroup analyses

The lack of interaction between SBP/DBP and relevant subgroups indicates that the significant prognostic value of SBP/DBP is consistent across various patient statuses. However, the authors observed a significant interaction between etiology and RHR for the prediction of all-cause mortality, with RHR influencing outcomes mainly on organic TR but with no effect on functional TR. In addition, a significant interaction between beta-blocker usage and RHR for the prediction of all-cause mortality was observed. The prognostic value of RHR was significant for patients with TR not using beta-blockers and invalid for those using these medications. The heterogeneity within beta-blocker subgroups may be interpreted as reduced RHR due to medications in patients with TR at a high risk of death. However, the mechanisms underlying the heterogeneity of prognostic value require further studies.

### Clinical implication

Similar to previous studies, the proportion of TR interventions during follow-up in this study was relatively low. The inadequacy of interventions is closely related to controversies concerning their management. These controversies include high operative mortality associated with isolated tricuspid valve surgery, (31) uncertain surgical benefit, (32) and indefinite surgical indications (33, 34). Therefore, it is important to identify patients with a high-risk of TR for close observation and careful management.

Clinically, a combination of these routinely measured indicators provides incremental prognostic value, in addition to baseline characteristics. Therefore, we propose that patients having TR with SBP <120 mmHg, DBP <70 mmHg, and RHR ≥80 bpm should be given more attention, further evaluation, and close follow-up in clinical practice. In addition, in the follow-up of patients with TR, when there is a decrease in SBP, DBP or an increase in RHR, it may indicate that the patient has changed hemodynamics or may have a high risk of death. For patients with similar symptoms of cardiac function, baseline BP or RHR can be used as hemodynamic markers for predicting adverse outcomes, which may help identify high-risk patients and provide further evaluation or intervention.

### Limitations

First, although independent associations of SBP, DBP, and RHR with all-cause mortality were observed in this study, the study's aim was not to elucidate mechanistic insights which how BP and RHR contribute to the worse prognosis of patients with TR. In a strict sense, the inherent limitations of observational studies do not support causal inferences between these hemodynamic parameters and all-cause mortality. Further physiological studies are required to confirm these hypotheses. Second, the endpoint of the current study was all-cause mortality, which was the most significant outcome, instead of cardiac death or major adverse cardiovascular events. Third, we could not collect detailed tricuspid- or right-heart-specific echocardiographic data from all centers due to the limitations of routine clinical echocardiography. Fourth, we only assessed patients for all-cause mortality based on baseline BP and RHR instead of time-averaged values during the entire follow-up period. Robust standardization and multiple modalities for measuring BP and RHR may provide more valuable results and meaningful clinical implications. Finally, since this study only included the Chinese population, caution should be exercised when generalizing our findings to other ethnicities.

## Conclusion

This national, multicenter, prospective cohort study demonstrated that SBP, DBP, and RHR are associated with all-cause mortality in patients with TR; lower SBP (<120 mmHg), lower DBP (<70 mmHg), and higher RHR (≥80 bpm) are prognostic factors independent of demographics, comorbidities, echocardiographic indices, medications, and serological tests, and etiologies. These routinely and clinically used markers, which are widely measured and easily interpreted by primary care practitioners and specialist doctors, may provide valuable and feasible indicators to identify patients with TR at a high risk of death.

However, given several potential limitations of this study, cautions need to be taken when generalizing our findings to routine clinical practice. Moreover, further large-scale and high-quality cohort studies are required to validate our findings.

## Data availability statement

The raw data supporting the conclusions of this article will be made available by the authors, without undue reservation.

## Ethics statement

The studies involving human participants were reviewed and approved by Ethics Committee of Fuwai Hospital. The patients/participants provided their written informed consent to participate in this study.

## Author contributions

YW, HX, and RG conceived the study. SG and BZ designed the study, conducted the statistical analyses, interpreted the results, and edited the first draft of the manuscript. YY, ZL, QL, ZZ, WW, ZY, HZ, QZ, ZD, JL, EZ, BW, and YZ were involved in the data collection and audit. YW and HX revised the manuscript accordingly. All authors contributed to the manuscript and approved the submitted version.

## Funding

This study was supported by the Chinese Academy of Medical Sciences Innovation Fund for Medical Sciences (Grant Number: 2017-12M-3-002).

## Conflict of interest

The authors declare that the research was conducted in the absence of any commercial or financial relationships that could be construed as a potential conflict of interest.

## Publisher's note

All claims expressed in this article are solely those of the authors and do not necessarily represent those of their affiliated organizations, or those of the publisher, the editors and the reviewers. Any product that may be evaluated in this article, or claim that may be made by its manufacturer, is not guaranteed or endorsed by the publisher.

## Abbreviations

DBP: diastolic blood pressure
eGFR: estimated glomerular filtration rate
Lai: indexed left atrial end-diastolic dimension
LVEDDi: indexed left ventricular end-diastolic dimension
LVEF: left ventricular ejection fraction
RHR: resting heart rate
SBP: systolic blood pressure
TR: tricuspid regurgitation.
