# Supplementary material for: Prognostic value of blood pressure and resting heart rate in patients with tricuspid regurgitation
Source: Front Cardiovasc Med. 2022 Aug 3;9:937412. doi: 10.3389/fcvm.2022.937412 (PMC9385404; doi:10.3389/fcvm.2022.937412)
Supplement: Supplementary file 1 [file Table_1.docx]

**Supplementary Table 1. Incremental Prognostic Value of SBP, DBP, and RHR**

| Models | C-Statistic (95% CI) | Change in C-Statistic (95% CI) | p Value | IDI (95% CI) | p Value | NRI (95% CI) | p Value |
| --- | --- | --- | --- | --- | --- | --- | --- |
| Model 1. Age, sex, BMI, Diabetes, CAD, PH, eGFR, symptoms, severe TR, LVEF, LVEDDi | 0.740(0.701-0.780) | - | - | - | - | - |  |
| Model 2. Model 1 + SBP | 0.750(0.713-0.788) | 0.009(-0.002-0.022) | 0.105 | 0.007(-0.001-0.024) | 0.066 | 0.100(-0.008-0.181) | 0.068 |
| Model 3. Model 1 + DBP | 0.753(0.714-0.791) | 0.012(-0.002-0.026) | 0.095 | 0.008(0.000-0.026) | 0.056 | 0.104(0.011-0.197) | 0.038 |
| Model 4. Model 1 + RHR | 0.749(0.712-0.787) | 0.009(-0.004-0.020) | 0.150 | 0.005(-0.001-0.019) | 0.140 | 0.140(0.006-0.217) | 0.038 |
| Model 5. Model 1 + SBP, DBP, RHR | 0.768(0.732-0.804) | 0.027(0.003-0.049) | 0.020 | 0.021(0.006-0.049) | 0.004 | 0.179(0.056-0.262) | 0.006 |

BMI: body mass index; CAD: coronary heart disease; DBP: diastolic blood pressure; eGFR: estimated glomerular filtration rate; LVEDDi: indexed left ventricular end-diastolic dimension; LVEF: left ventricular ejection fraction; PH: pulmonary hypertension; RHR: resting heart rate; SBP: systolic blood pressure; TR: tricuspid regurgitation.
