# Supplementary figures and images for: Prognostic value of blood pressure and resting heart rate in patients with tricuspid regurgitation
Source: Front Cardiovasc Med. 2022 Aug 3;9:937412. doi: 10.3389/fcvm.2022.937412 (PMC9385404; doi:10.3389/fcvm.2022.937412)

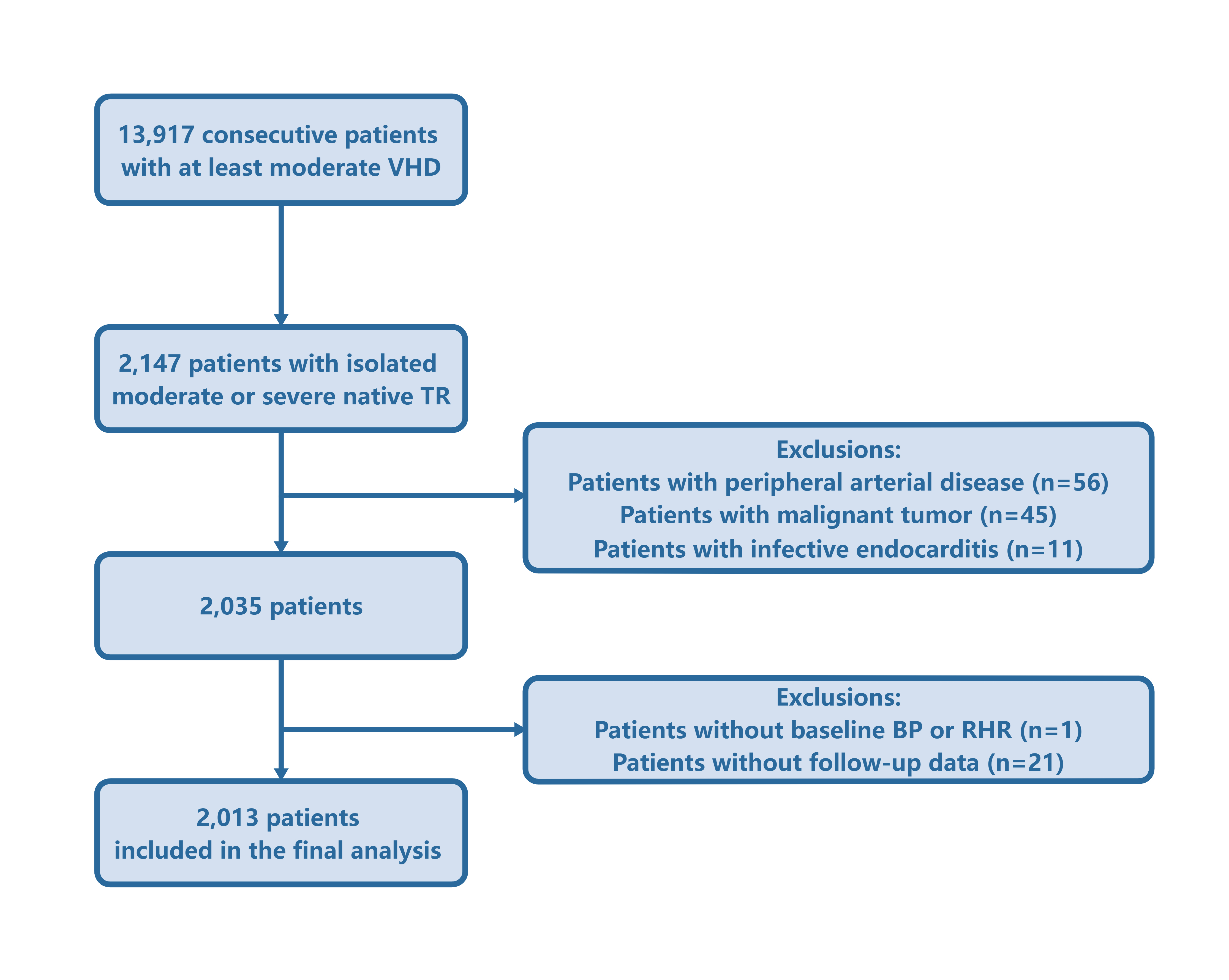

Supplement: Supplementary file 3 [file Image_1.JPEG]
